# Psychometric properties and general population reference values for PROMIS Global Health in Hungary

**DOI:** 10.1007/s10198-023-01610-w

**Published:** 2023-06-28

**Authors:** Alex Bató, Valentin Brodszky, Ariel Zoltán Mitev, Balázs Jenei, Fanni Rencz

**Affiliations:** 1 Károly Rácz Doctoral School of Clinical Medicine, Semmelweis University, Budapest, Hungary; 2 Department of Health Policy, Corvinus University of Budapest, 8 Fővám tér, Budapest, 1093 Hungary; 3 Institute of Marketing and Communication Sciences, Corvinus University of Budapest, Budapest, Hungary

**Keywords:** Hungary, Generic health status measures, Item response theory, PROMIS, Population norm, Psychometrics, I10

## Abstract

**Objectives:**

Patient-Reported Outcomes Measurement Information System–Global Health (PROMIS-GH) is a widely used generic measure of health status. This study aimed to (1) assess the psychometric properties of the Hungarian PROMIS-GH and to (2) develop general population reference values in Hungary.

**Methods:**

An online cross-sectional survey was conducted among the Hungarian adult general population (*n* = 1700). Respondents completed the PROMIS-GH v1.2. Unidimensionality (confirmatory factor analysis and bifactor model), local independence, monotonicity (Mokken scaling), graded response model fit, item characteristic curves and measurement invariance were examined. Spearman’s correlations were used to analyse convergent validity of PROMIS-GH subscales with SF-36v1 composites and subscales. Age- and gender-weighted T-scores were computed for the Global Physical Health (GPH) and Global Mental Health (GMH) subscales using the US item calibrations.

**Results:**

The item response theory assumptions of unidimensionality, local independence and monotonicity were met for both subscales. The graded response model showed acceptable fit indices for both subscales. No differential item functioning was detected for any sociodemographic characteristics. GMH T-scores showed a strong correlation with SF-36 mental health composite score (*r*_s_ = 0.71) and GPH T-scores with SF-36 physical health composite score (*r*_s_ = 0.83). Mean GPH and GMH T-scores of females were lower (47.8 and 46.4) compared to males (50.5 and 49.3) (*p* < 0.001), and both mean GPH and GMH T-scores decreased with age, suggesting worse health status (*p* < 0.05).

**Conclusion:**

This study established the validity and developed general population reference values for the PROMIS-GH in Hungary. Population reference values facilitate the interpretation of patients’ scores and allow inter-country comparisons.

**Supplementary Information:**

The online version contains supplementary material available at 10.1007/s10198-023-01610-w.

## Introduction

Health status measures are widely used in clinical practice, observational studies, clinical trials, monitoring general population health, assessing the performance of health systems and in cost-effectiveness analysis [1]. Two forms of health status measures can be distinguished as follows: condition-specific and generic [2]. Condition-specific measures have a specific target population and are able to capture a wide range of symptoms and health problems relevant to a certain condition (e.g. itching in skin diseases or bowel problems in gastrointestinal diseases). In contrast, generic health status measures incorporate health areas that are relevant across different patient populations as well as for the general public (e.g. physical functioning, pain, sleeping). These measures have the advantage of allowing comparisons across different conditions, health interventions and with general population reference values.

In general, a large number of items are needed to precisely assess one’s health status; however, this may lack practical considerations (e.g. time and respondent burden). Therefore, short-form health assessments have gained popularity. Commonly used short generic health status measures include the EQ-5D and SF-36 [3, 4]. These instruments, however, were developed decades ago and one of their common criticisms is that their item development and selection did not benefit from modern psychometric methods, such as item response theory (IRT). The Patient Reported Outcomes Measurement Information System (PROMIS) initiative, funded by the National Institutes of Health in the US, aimed to develop, validate and standardize item banks to measure health outcomes across a broad range of health areas [5]. In the past two decades, over 100 PROMIS item banks and a few fixed-length short-forms have been developed using IRT methods (e.g. PROMIS Global Health, PROMIS-29, PROMIS-43, PROMIS-57) [6, 7]. The main advantages of IRT over classical test theory methods include the estimation of the respondents’ location on an underlying ‘latent’ trait (e.g. health status) based on any subset of items that do not vary depending on the characteristics of the population and the possibility to adaptively assess health status using computerised adaptive testing [8, 9].

PROMIS Global Health (PROMIS-GH) is the shortest PROMIS short-form that measures five generic domains of health (physical functioning, pain, fatigue, emotional distress, social health) using 10 global health items [10]. Its validity, reliability and responsiveness have been confirmed in several populations, including patients with stroke [11], orthopaedic conditions [12–16], amyloidosis [17], inflammatory bowel diseases [18], pregnant women [19] and older adults [20]. The international use of PROMIS-GH has also been expanding outside the US, including studies from the UK [21], Germany [22] and the Netherlands [23]. Furthermore, two countries, the US and the Netherlands have also established general population reference values [24, 25]. So far, PROMIS-GH has not been used in Hungary. Therefore, this study aimed to evaluate the psychometric performance of the Hungarian PROMIS-GH and to develop general population reference values in Hungary.

## Methods

### Study design and recruitment

The study was approved by the Research Ethics Committee of the Corvinus University of Budapest (no. KRH/343/2020). In November 2020, an online cross-sectional survey was conducted among the Hungarian adult general population. Respondents were recruited by a survey company from members of the largest Hungarian online panel. ‘Soft quotas’ were used for age, gender, education, place of living and geographical region to approximate the distribution of the general population. The inclusion criteria for this study were as follows: (i) ≥ 18 years of age; (ii) place of residence in Hungary; and (iii) giving informed consent prior to data collection.

PROMIS-GH v1.2 was administered as part of a longer survey that aimed to assess the health status and well-being among members of the general public in Hungary [26–28]. Respondents were also asked to complete the SF-36v1 and to identify their sociodemographic background (gender, age, education, place of residence, region, employment, household’s net monthly income, marital status, body weight and height) and if they had any chronic health conditions. All respondents first completed SF-36, followed by PROMIS-GH.

### Measures

#### PROMIS Global Health (PROMIS-GH)

The official Hungarian version of PROMIS-GH v1.2 was used as provided by the PROMIS Health Organization. PROMIS-GH consists of 10 items, namely Global01 = general health, Global02 = quality of life, Global03 = physical health, Global04 = mental health, Global05 = satisfaction with discretionary social activities, Global06 = physical function, Global07 = pain, Global08 = fatigue, Global09 = social roles and Global10 = emotional problems [10]. It has two subscales, Global Physical Health (GPH) and Global Mental Health (GMH). GPH consists of Global03, Global06, Global07 and Global08, while GMH includes Global02, Global04, Global05 and Global10. The recall period of the items varies across ‘in general’, the ‘past seven days’ and unspecified. Each item is assessed on a scale with five response levels. For Global01, Global02, Global03, Global04, Global05 and Global09, the best response option is excellent (5), and the worst is poor (1). For Global06 options range from completely (5) to not at all (1), for Global10 from never (5) to always (1) and for Global08 from none (5) to very severe (1). An exception is Global07, which is rated from 0 to 10 (0 = no pain, 10 = worst imaginable pain). We recoded Global07 to a 5-point scale as follows: 0 = 5; 1–3 = 4; 4–6 = 3; 7–9 = 2; 10 = 1 [10]. Raw subscale scores were calculated by adding scores of individual items per subscale. We calculated standardized T-scores from raw scores using the US item calibrations [29]. Mean T-scores therefore represent the mean of the US general population. A higher T-score indicates better health status and a lower T-score refers to worse health status compared to the US general population, where the general population mean is set at 50 with a standard deviation of 10 [24].

#### 36-item short form health survey (SF-36)

The Hungarian version of the SF-36v1 questionnaire was used in our survey with a 4-week recall period. SF-36 is a generic health status measure with 36 items that cover eight health subscales, specifically (1) physical functioning, (2) role limitations due to physical problems (3) bodily pain, (4) general health, (5) vitality, (6) social functioning, (7) role limitations due to emotional problems and (8) mental health [4, 30]. Responses to items are transformed to range from 0 to 100, where higher scores represent better health status. Subscale scores are computed by averaging the respective item scores. SF-36 allows the generation of two summary scores, one for physical health (physical health composite) that includes the first four subscales (1–4) and the other for mental health (mental health composite) including the last four (5–8).

### Statistical analyses

In this study, we built on the methods used in earlier psychometric investigations and reference population studies with PROMIS instruments [9, 10, 23, 25]. Data analysis was carried out in R Statistical Software (v4.1.2 Vienna, Austria). We used both classical test theory (e.g. ceiling and floor effect, convergent validity, factor analysis) and IRT methods. Before IRT modelling, we tested the following three assumptions: unidimensionality, local independence and monotonicity [31]. In addition, differential item functioning (DIF) analysis was used to examine measurement invariance. Raw item and subscale scores were used to analyse ceiling and floor effect and for the factor analysis, IRT and DIF analyses. Unweighted T-scores were used to draw histograms and estimate correlations. T-scores were weighted for age group and gender to calculate Hungarian GPH and GMH general population reference values.

#### Ceiling and floor effect

Ceiling and floor effect were considered if GPH and GMH raw subscale scores exceeded 15% [32].

#### Unidimensionality

Unidimensionality was tested using confirmatory factor analysis (CFA) and bifactor models. CFA was conducted for the two subscales separately (*lavaan* package) [33]. Goodness-of-fit was evaluated by the comparative fit index (CFI, cut-off value: > 0.95), Tucker-Lewis index (TLI, cut-off value: > 0.95), the root mean square error of approximation (RMSEA, cut-off value < 0.06), and the standardized root mean squared residual (SRMR, cut-off value: < 0.08) [34, 35]. Further, we used bifactor models to obtain Omega Hierarchical (tentative benchmark > 0.70) and explained common variance (ECV, tentative benchmark > 0.60) [36, 37]. The bifactor models were developed using the *psych* package [38].

#### Local independence

To test local independence, we examined the residual correlation matrix resulting from the CFA for both GPH and GMH subscales. Residual correlation values between − 0.20 and 0.20 were considered acceptable supporting local independence [9].

#### Monotonicity

Monotonicity was analysed using Mokken scale analysis (mokken package). Coefficients (H_i_ for items, H for subscales) exceeding the cut-off value of > 0.30 were considered acceptable [39, 40].

#### IRT model fit

Given the polytomous response options of PROMIS-GH items, a graded response model was fitted for both GPH and GMH (mirt package) [41, 42]. To detect item misfit, we used Orlando and Thissen’s S-χ^2^. Items with *p*-value < 0.001 were considered misfitting [43]. The same cut-off values were used for fit indices (CFI, TLI, RMSEA, SRMR) as for unidimensionality [34]. Item discrimination (slope, a) and item difficulties (threshold, b) were also computed. Item characteristic curves (ICC) were generated for each item of the two subscales.

#### Measurement invariance

Measurement invariance was assessed by analysing differential item functioning (DIF) using the *lordif *package [44]. DIF occurs when the responses of a subgroup of respondents on an item consistently differ from those of another subgroup when controlling for the underlying level of the trait measured by the scale [9]. DIF was analysed for GPH and GMH with the following subgroups: gender (female, male), median age (< 47, ≥ 47 years), education (primary, secondary, tertiary), region (Central, Western and Eastern Hungary), employment (employed, not employed), place of residence (capital, other town, village), marital status (married, not married), and income groups (quintiles, do not know, refused to answer groups). First, we used ordinal logistic regression models without an anchor to evaluate DIF. Where DIF was detected, we repeated the analysis using non-DIF items as an anchor. A Pseudo *R*^2^ change ≥ 0.02 was taken as a critical value [45, 46]. The details of the DIF analysis are provided elsewhere [28].

#### Convergent validity

Spearman’s rank order correlations were used to explore the convergent validity of the two PROMIS-GH subscales with the eight SF-36 subscales and two composite scores. Correlation coefficients (*r*_s_) were interpreted as very weak (< 0.20), weak (0.20–0.39), moderate (0.40–0.59) and strong correlation (0.60 ≤) [47].

#### Establishment of general population reference values

Mean GPH and GMH T-scores were weighted according to gender and age group to derive general population reference values using the US item calibrations [48]. Mean weighted T-scores were computed for subgroups of respondents defined by gender, age groups, education, place of residence, region, employment, income groups, marital status, health status question of SF-36 (item 1), BMI and the presence of any chronic condition. We used Taylor linearization for standard errors, and 95% confidence intervals were calculated for each group. The subgroups were compared using Mann–Whitney or Kruskal–Wallis tests, where applicable.

#### Hypotheses

Regarding the psychometric properties, we hypothesized (1) no ceiling or floor effects for any subscales, (2) unidimensionality, (3) local independence, (4) monotonicity, (5) acceptable fit to the graded response model, (6) no measurement invariance for any subgroups, (7) moderate or strong correlations between the PROMIS-GH subscales (GPH and GMH) and their corresponding SF-36 composite scores [10, 11, 23, 49]. With regard to the reference values, we hypothesized better self-reported health in men and declining physical health with age [50].

## Results

### Sample characteristics (unweighted)

Overall, 2502 respondents initiated the survey, 2079 of whom consented and 379 quit before the end of the questionnaire. A total of 1700 respondents completed the survey. The mean age was 47.9 ± 16.3 years, and 56.3% of the respondents were female. Nearly one-third of the sample had tertiary education (32.4%). Half of the respondents were employed (50.9%), 23.5% were retired and 4.4% were students. Overall, 22.4% lived in the capital, 48.2% in other towns and 29.4% in villages. The geographical distribution of the sample was as follows: Western Hungary 29.0%, Central Hungary 33.6%, Eastern Hungary 37.4%. Overall, 67.4% of the sample reported to have any chronic disease. The overall sample showed a good representativeness for the general population in Hungary; however, respondents with a secondary education were slightly underrepresented and those who lived in the capital were somewhat overrepresented (Table 1).

**Table 1:** Characteristics of the study population and PROMIS Global Health reference values in Hungary

Variables	General population [46]	Unweighted sample	Weighted sample	Global Physical Health (GPH) T-score					Global Mental Health (GMH) T-score				
	%	*N* (%)	*N* (%)	Mean	Confidence interval (95%)	Median	IQR	*p*-value	Mean	Confidence interval (95%)	Median	IQR	*p*-value^a^
Total	–	1700	1700	49.04	48.60–49.49	47.7	42.3–54.1	–	47.7	47.27–48.22	48.3	41.1–53.3	–
Gender													
Female	53.1	957 (56.3)	902 (53.1)	47.75	47.14–48.35	47.7	42.3–54.1	< 0.001	46.41	45.79–47.02	45.8	41.1–53.3	< 0.001
Male	46.9	743 (43.7)	798 (46.9)	50.50	49.85–51.16	50.8	44.9–57.7		49.26	48.51–50.00	48.3	43.5–56.0	
Age groups (years)													
18–24	10.0	148 (8.7)	169 (9.9)	52.28	50.63–53.92	50.8	47.7–57.7	< 0.001	49.87	47.84–51.90	48.3	43.5–56.0	0.011
25–34	15.2	293 (17.2)	259 (15.2)	50.93	49.91–51.94	50.8	44.9–57.7		49.26	47.98–50.54	48.3	43.5–56.0	
35–44	19.5	309 (18.2)	331 (19.5)	49.96	48.99–50.92	50.8	44.9–54.1		48.20	47.07–49.32	48.3	41.1–56.0	
45–54	16.0	304 (17.9)	272 (16.0)	48.71	47.69–49.73	50.8	42.3–54.1		47.23	46.18–48.28	48.3	41.1–53.3	
55–64	16.8	296 (17.4)	286 (16.8)	47.68	46.59–48.77	47.7	42.3–54.1		46.34	45.33–47.34	45.8	41.1–53.3	
65 +	22.5	350 (20.6)	383 (22.5)	46.80	45.79–47.81	47.7	39.8–54.1		46.80	45.86–47.73	45.8	41.1–53.3	
Highest level of education													
Primary school or less	23.8	468 (27.5)	464 (27.3)	47.05	46.00–48.09	47.7	39.8–54.1	< 0.001	46.37	45.33–47.41	45.8	38.8–53.3	< 0.001
Secondary school	55.0	682 (40.1)	692 (40.7)	49.09	48.43–49.76	47.7	44.9–54.1		47.34	46.59–48.09	45.8	41.1–53.3	
College/university degree	21.2	550 (32.4)	544 (32.0)	50.68	49.97–51.38	50.8	44.9–57.7		49.42	48.67–50.18	48.3	43.5–56.0	
Place of residence													
Capital	17.9	380 (22.4)	384 (22.6)	49.53	48.65–50.41	50.8	44.9–54.1	< 0.001	47.99	47.08–48.90	48.3	43.5–53.3	< 0.001
Other town	52.6	820 (48.2)	814 (47.9)	49.89	49.23–50.56	50.8	44.9–57.7		48.54	47.83–49.26	48.3	43.5–56.0	
Village	29.5	500 (29.4)	503 (29.6)	47.29	46.44–48.13	47.7	39.8–54.1		46.26	45.38–47.14	45.8	38.8–53.3	
Geographical region													
Central Hungary	30.4	572 (33.6)	581 (34.2)	49.38	48.65–50.11	50.8	42.3–54.1	0.203	47.87	47.09–48.64	48.3	43.5–43.3	0.175
Western Hungary	30.2	493 (29.0)	480 (28.2)	48.61	47.83–49.40	47.7	42.3–54.1		47.29	46.43–48.15	45.8	41.1–53.3	
Eastern Hungary	39.5	635 (37.4)	640 (37.6)	49.20	48.33–50.06	50.8	42.3–54.1		48.20	47.33–49.07	48.3	43.5–56.0	
Employment status													
Employed	53.1	865 (50.9)	858 (50.5)	50.28	49.71–50.85	50.8	44.9–54.1	< 0.001	48.77	48.12–49.41	48.3	43.5–56.0	< 0.001
Retired	26.1	399 (23.5)	427 (25.1)	46.87	45.91–47.83	47.7	39.8–54.1		46.89	46.03–47.76	45.8	41.1–53.3	
Disability pensioner	3.1	67 (3.9)	64 (3.8)	39.79	37.44–42.14	39.8	32.4–47.7		41.04	38.49–43.59	38.8	33.8–48.3	
Student	3.1	74 (4.4)	87 (5.1)	53.59	51.60–55.58	54.1	47.7–57.7		50.17	47.19–53.16	48.3	43.5–56.0	
Unemployed	4.7	129 (7.6)	125 (7.4)	49.88	47.81–51.94	50.8	42.3–57.7		46.43	44.25–48.61	45.8	38.8–56.0	
Homemaker/housewife	1.0	99 (5.8)	80 (4.7)	48.86	47.38–50.33	47.7	44.9–54.1		47.18	45.19–49.17	48.3	41.1–53.3	
Other	0.0	67 (3.9)	60 (3.5)	48.62	46.30–50.95	47.7	42.3–57.7		46.26	43.65–48.88	45.8	41.1–53.3	
Household net monthly income per person (HUF)^b^													
First quintile (0–66,779)	n/a	224 (13.2)	217 (12.8)	46.22	44.72–47.72	44.9	39.8–54.1	< 0.001	43.60	42.06–45.14	43.5	36.3–50.8	< 0.001
Second quintile (66,780–99,511)		252 (14.8)	255 (15.0)	46.66	45.37–47.94	47.7	39.8–54.1		45.92	44.55–47.29	45.8	38.8–53.3	
Third quintile (99,512–126,924)		229 (13.5)	234 (13.8)	49.59	48.26–50.93	50.8	42.3–57.7		48.44	47.09–49.78	48.3	43.5–56.0	
Fourth quintile (126,925–164,049)		207 (12.2)	207 (12.2)	48.89	47.72–50.06	47.7	42.3–54.1		48.11	46.84–49.39	48.3	43.5–53.3	
Fifth quintile (164,050-)		423 (24.9)	425 (25.0)	50.47	49.67–51.27	50.8	44.9–54.1		49.55	48.73–50.37	48.3	43.5–56.0	
Do not know		69 (4.1)	74 (4.4)	51.56	49.25–53.87	50.8	44.9–57.7		50.05	47.40–52.69	48.3	43.5–56.0	
Do not want to answer		296 (17.4)	288 (16.9)	50.19	49.21–51.17	50.8	44.9–54.1		48.39	47.33–49.45	48.3	43.5–53.3	
Marital status													
Married	45.6	718 (42.2)	691 (40.6)	48.74	48.05–49.43	50.8	42.3–54.1	0.038	48.40	47.67–49.13	48.3	43.5–53.3	0.221
Domestic partnership	13.4	360 (21.2)	348 (20.5)	49.02	48.12–49.92	47.7	42.3–54.1		47.75	46.77–48.73	48.3	41.1–53.3	
Single	18.5	336 (19.8)	350 (20.6)	50.58	49.51–51.64	50.8	44.9–57.7		47.12	45.88–48.36	45.8	41.1–53.3	
Widowed	11.4	98 (5.8)	115 (6.8)	47.23	45.27–49.18	44.9	39.8–54.1		46.94	45.26–48.61	45.8	43.5–53.3	
Divorced	11.1	156 (9.2)	163 (9.6)	48.52	46.96–50.07	47.7	39.8–57.7		46.70	45.13–48.27	48.3	38.8–53.3	
Other	n/a	32 (1.9)	33 (1.9)	48.20	44.70–51.70	50.8	42.3–57.7		48.43	44.38–52.48	45.8	43.5–56.0	
Self-perceived health status (first question of SF-36)													
Excellent	n/a	139 (8.2)	145 (8.5)	60.47	58.99–61.95	61.9	57.7–67.7	< 0.001	59.40	57.62–61.18	62.5	56.0–67.6	< 0.001
Very good		401 (23.6)	412 (24.2)	55.33	54.68–55.98	54.1	50.8–57.7		53.39	52.60–54.18	53.3	48.3–56.0	
Good		682 (40.1)	668 (39.3)	49.13	48.68–49.57	47.7	44.9–54.1		46.80	46.27–47.34	45.8	43.5–50.8	
Fair		388 (22.8)	386 (22.7)	41.76	41.20–42.33	42.3	37.4–44.9		42.21	41.45–42.97	41.1	36.3–45.8	
Poor		90 (5.3)	90 (5.3)	32.51	31.34–33.68	32.4	29.6–34.9		33.91	32.32–35.50	33.8	28.4–38.8	
BMI groups													
Underweight (under 18.5)	n/a	56 (3.3)	49 (2.9)	49.71	47.27–52.15	50.8	44.9–54.1	< 0.001	47.09	44.30–49.87	45.8	41.1–53.3	0.075
Normal weight (between 18.5 and 24.9)		497 (29.2)	500 (29.4)	51.01	50.16–51.86	50.8	44.9–57.7		48.27	47.33–49.21	48.3	43.5–56.0	
Overweight (between 25 and 29.9)		535 (31.5)	539 (31.7)	49.58	48.80–50.36	50.8	42.3–54.1		48.21	47.37–49.06	48.3	41.1–53.3	
Obesity (between 30 and 39.9)		374 (22.0)	375 (22.1)	46.61	45.70–47.52	47.7	39.8–54.1		46.79	45.83–47.74	45.8	41.1–53.3	
Chronic disease^c^													
Yes	48.0	1146 (67.4)	1127 (66.3)	46.72	46.20–47.24	47.7	39.8–54.1	< 0.001	45.91	45.35–46.47	45.8	38.8–53.3	< 0.001
No	52.0	410 (24.1)	423 (24.9)	54.66	53.81–55.50	54.1	50.8–61.9		52.38	51.44–53.32	53.3	45.8–59.0	
Do not know/want to answer	–	144 (8.5)	150 (8.8)	50.65	49.20–52.11	50.8	44.9–57.7		48.48	56.71–50.24	48.3	43.5–56.0	

*BMI* Body mass index (*N* = 238 were missing, *p*-value was computed without these respondents), *HUF* Hungarian forint, *IQR* interquartile range, *n/a* not available

^a^Computed by Mann–Whitney and Kruskal–Wallis tests

^b^*p*-values were calculated after excluding the ‘I do not know’ and ‘I do not want to answer’ responses

^c^Hungarian Central Statistical Office, Health at a Glance 2019

### Ceiling and floor effect

**Fig. 1:**
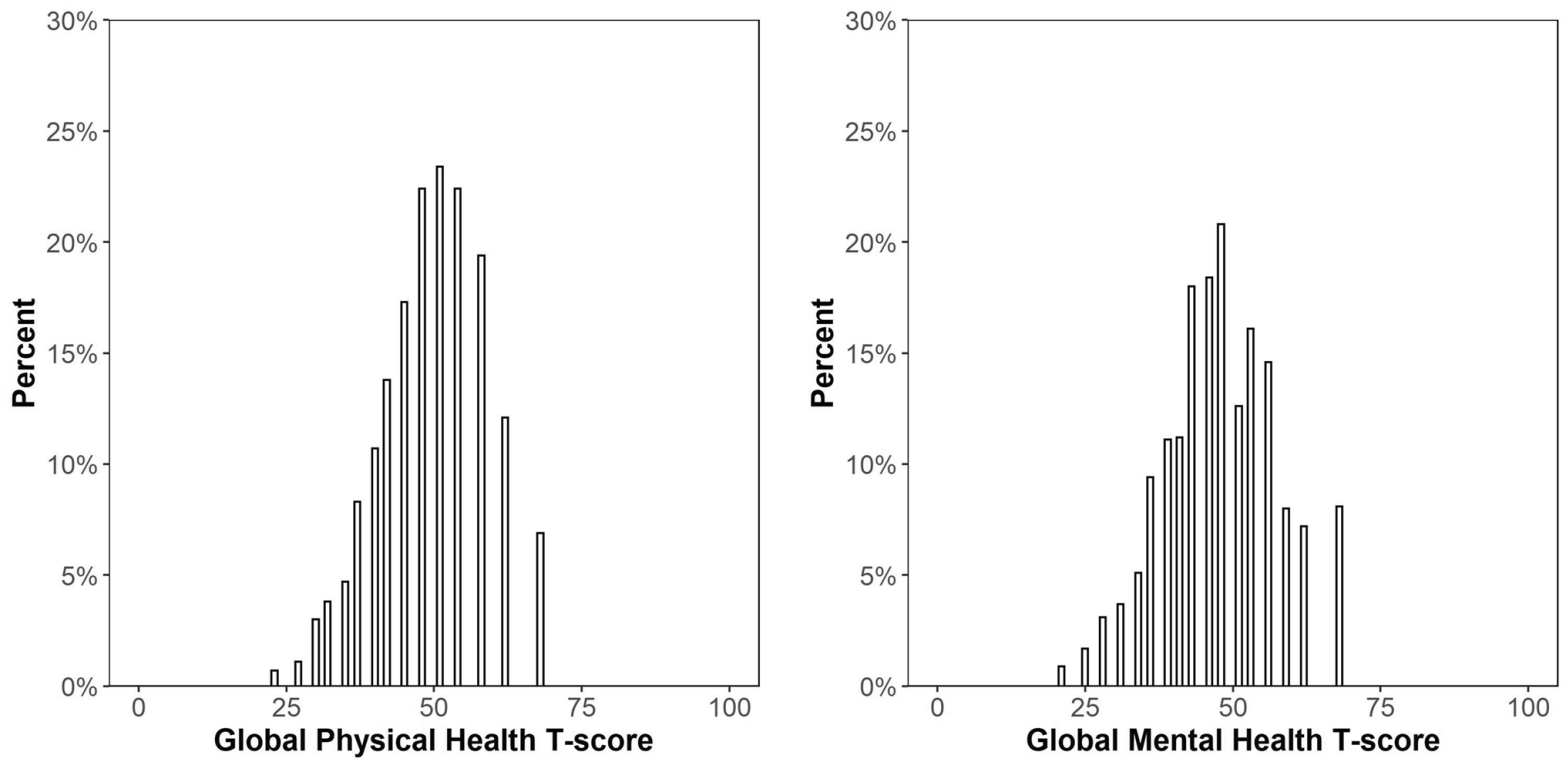
Distribution of Global Physical Health and Global Mental Health T-scores (unweighted)

The distributions of GPH and GMH raw scores are presented in Fig. 1. We found almost no floor and low ceiling effect for both GPH (0.4% and 4.1%) and GMH subscales (0.5% and 4.8%) (Table 2). Among the items, Global07 demonstrated the highest floor (29.8%). Global06 showed the highest ceiling (58.2%), followed by Global10 (38.3%), Global08 (23.9%) and Global09 (15.8%).

**Table 2:** Floor and ceiling of PROMIS Global Health items and subscales

Items and subscales	Floor^a^		Ceiling^b^	
	*n*	%	*n*	%
Global01 (general health)	89	5.24	175	10.29
Global02 (quality of life)	81	4.76	162	9.53
Global03 (physical health)	107	6.29	156	9.18
Global04 (mental health)	95	5.59	252	14.82
Global05 (satisfaction with discretionary social activities)	107	6.29	245	14.41
Global06 (physical function)	27	1.59	990	58.24
Global07 (0–10 pain intensity numeric rating scale)^c^	507	29.82	5	0.29
Global08 (fatigue)	17	1.00	407	23.94
Global09 (social roles)	67	3.94	269	15.82
Global10 (emotional problems)	40	2.35	651	38.29
Global Physical Health (GPH)	7	0.41	69	4.06
Global Mental Health (GMH)	9	0.53	81	4.76

^a^Worst health status for all items except for Global07

^b^Best health status for all except for Global07

^c^Not reverse coded item

### Factor and IRT analysis

#### Unidimensionality

Fit indices confirmed the unidimensionality of both GPH (CFI = 0.993, TLI = 0.978, SRMR = 0.039) and GMH (CFI = 0.999, TLI = 0.997, SRMR = 0.025), with the exception of RMSEA (GPH 0.114 and GMH 0.071). The hypotheses were supported by the bifactor models, resulting in ECV values higher than the tentative benchmark for both subscales (GPH 0.72 and GMH 0.78). Omega Hierarchical was above the tentative benchmark only for GMH (0.73), but not for GPH (0.66) (Table 3).

**Table 3:** Psychometric properties of PROMIS Global Health subscales

	Unidimensionality		Monotonicity		Graded response model Item discrimination, difficulties and model fit indices											
	Omega hierarchical	ECV	*H*_i_ (SE)	*H* (SE)	*a*	*b*1	*b*2	*b*3	*b*4	Average *b*	*S*-*χ*2	*p*-value	RMSEA	SRMR	TLI	CFI
Global Physical Health (GPH)	0.66	0.72	–	0.531 (0.016)	–	–	–	–	–	–	–	–	0.008	0.045	0.905	0.968
Global03	–	–	0.541 (0.017)	–	2.045	– 2.053	– 0.697	0.646	1.740	– 0.091	88.703	< 0.001	–	–	–	–
Global06	–	–	0.552 (0.019)	–	2.143	– 2.879	– 2.177	– 1.071	– 0.252	– 1.595	52.425	< 0.001	–	–	–	–
Global07^c^	–	–	0.550 (0.017)	–	2.322	– 3.616	– 1.747	– 0.716	0.663	– 1.354	31.494	0.086	–	–	–	–
Global08	–	–	0.480 (0.019)	–	1.577	– 3.693	– 2.257	– 0.525	1.044	– 1.358	17.520	0.353	–	–	–	–
Global Mental Health (GMH)	0.73	0.78	–	0.638 (0.012)	–	–	–	–	–	–	–	–	0.012	0.031	0.969	0.990
Global02	–	–	0.600 (0.016)	–	2.059	– 2.179	– 0.885	0.544	1.721	– 0.200	74.081	< 0.001	–	–	–	–
Global04	–	–	0.717 (0.011)	–	8.005	– 1.601	– 0.697	0.193	1.066	– 0.260	32.060	0.002	–	–	–	–
Global05	–	–	0.657 (0.013)	–	2.898	– 1.771	– 0.799	0.299	1.242	– 0.257	69.617	< 0.001	–	–	–	–
Global10	–	–	0.571 (0.017)	–	1.721	– 2.925	– 1.628	– 0.655	0.420	– 1.197	116.608	< 0.001	–	–	–	–

*a* Item discrimination (slope), *b* item difficulty (threshold), *CFI* comparative fit index, *ECV* explained common variance, *Hi&H* Mokken scale analysis coefficients, *RMSEA* root mean square error of approximation, *SE* standard error, *SRMR* standardized root mean squared residual, *S-χ*2 item fit index, *TLI* Tucker-Lewis index

Global02 = quality of life, Global03 = physical health, Global04 = mental health, Global05 = satisfaction with discretionary social activities, Global06 = physical function, Global07 = pain (reverse coded 5-level item), Global08 = fatigue, Global10 = emotional problems

^c^Reverse coded item

#### Local independence

We found no local dependence between item pairs (Online Resource 1). Eight item pairs had negative residual correlations, but all values were above the value of − 0.20.

#### Monotonicity

The Mokken scale analysis resulted in coefficients higher than the cut-off value for both subscales (*H* = 0.531 and 0.638 for GPH and GMH) and items, ranging from *H*_i_ = 0.480 (Global08) to 0.717 (Global04) supporting monotonicity (Table 3).

#### Model fit

**Fig. 2:**
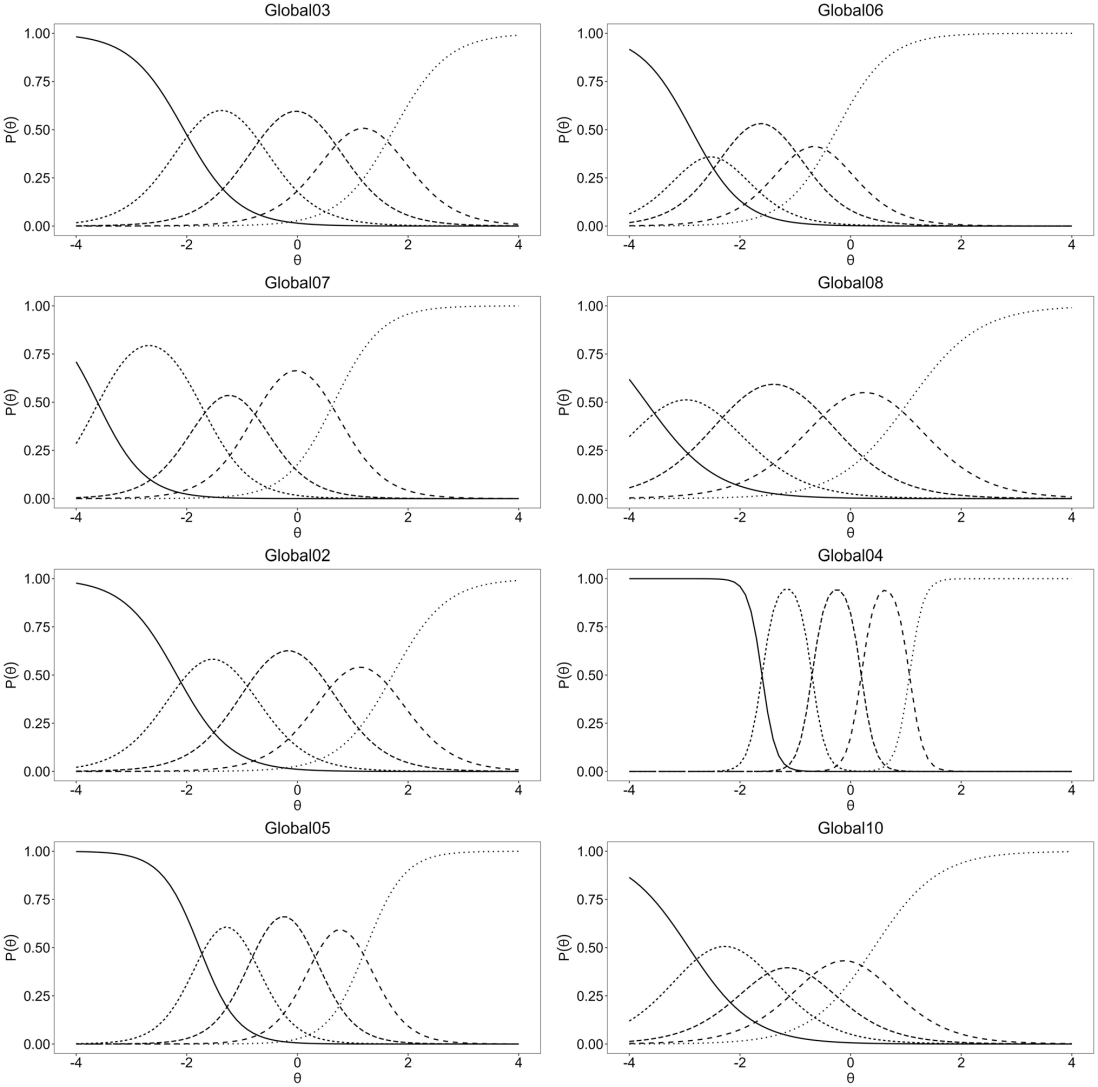
Item characteristic curves of items of the Global Physical Health and Global Mental Health subscales. Global02 = quality of life, Global03 = physical health, Global04 = mental health, Global05 = satisfaction with discretionary social activities, Global06 = physical function, Global07 = pain (reverse coded 5-level item), Global08 = fatigue, Global10 = emotional problems, Global Physical Health items: Global03, Global06, Global07, Global08. Global Mental Health items: Global02, Global04, Global05, Global10

Given that unidimensionality, local independence and monotonicity were supported for both subscales, graded response models were fitted. Acceptable fit indices were found for both subscales (GPH: RMSEA = 0.008, SRMR = 0.045, TLI = 0.905, CFI = 0.968 and GMH: RMSEA = 0.012, SRMR = 0.031, TLI = 0.969, CFI = 0.990). A few items showed misfit to the graded response model, namely Global03, Global06, Global02, Global05 and Global10 (*p* < 0.001) (Table 3). Item difficulties (b) ranged from − 3.7 (Global08) to 1.7 (Global03) for GPH and from − 2.9 (Global10) to 1.7 (Global02) for GMH. Item discrimination (a) values ranged from 1.6 (Global08) to 2.3 (Global07) and from 1.7 (Global10) to 8.0 (Global04) for GPH and GMH, respectively. ICCs for the two subscales are displayed in Fig. 2.

#### Measurement invariance

After the first step (without anchors), one item (Global07) was flagged for DIF based on age groups, and two items (Global02 and Global10) were flagged for DIF by gender. After the second step (with anchors), DIF was no longer detected for age group and gender, as the Pseudo *R*^2^ change was < 0.02 for each analysis. No DIF was detected for education, region, employment, place of residence, marital status or income at all.

#### Convergent validity

**Fig. 3:**
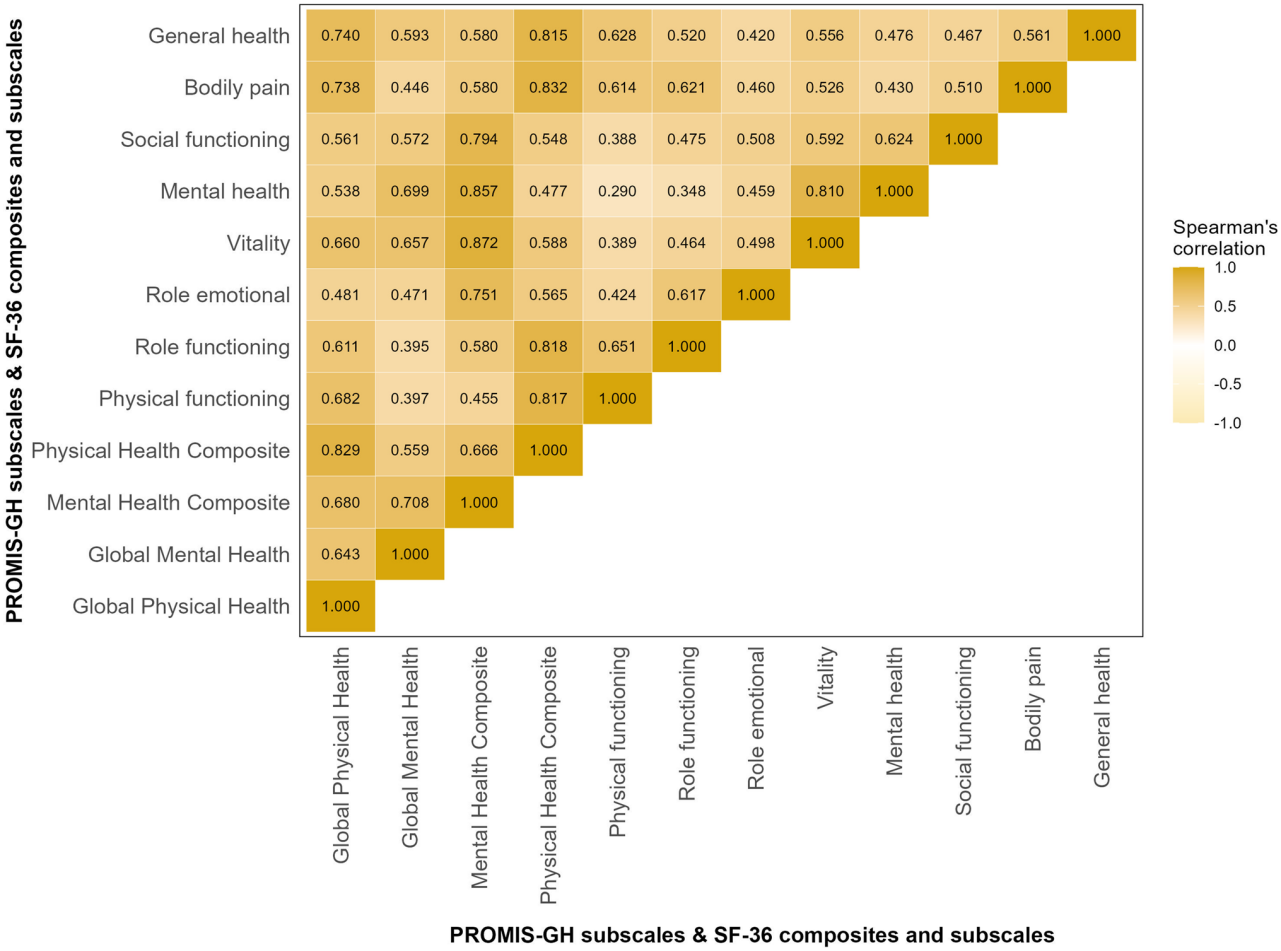
Convergent validity of PROMIS Global Health subscales with SF-36 composites and subscales. *p* < 0.001 for all correlation coefficients (Spearman’s). PROMIS-GH = Patient Reported Outcomes Measurement Information System-Global Health, SF-36 = 36-item short form health survey

GMH T-score showed a strong correlation with the mental health composite score of SF-36 (*r*_s_ = 0.708) and GPH T-score with the physical health composite score (*r*_s_ = 0.829) (Fig. 3). Among the SF-36 subscales, the GPH T-score had the highest correlation with general health (*r*_s_ = 0.740) and bodily pain (*r*_*s*_ = 0.738), while the GMH T-score showed the strongest correlation with mental health (*r*_s_ = 0.699) and vitality (*r*_s_ = 0.657).

#### Reference values for PROMIS-GH in Hungary

Mean total T-scores for GPH and GMH were 49.0 and 47.7, respectively (Table 1). Mean GPH and GMH T-scores of females were lower (47.8 and 46.4) compared to males (50.5 and 49.3) (*p* < 0.001). We found the highest mean T-scores for GPH and GMH in the 18–24 age group (GPH: 52.3 and GMH: 49.9). Mean GPH and GMH T-scores showed a decreasing trend with age (*p* < 0.05). Those with higher level of education, living in towns, being student, having higher income and without chronic disease had higher mean T-scores scores for both GPH and GMH (*p* < 0.001). With regard to BMI, mean GPH T-scores were higher in respondents with normal weight compared to those being underweight or overweight/obese (*p* < 0.05). Those who reported ‘excellent’ health on the first question of the SF-36 had the highest, while those who reported ‘poor’ had the lowest mean GPH and GMH T-scores (*p* < 0.001).

## Discussion

This study provided a psychometric assessment of the Hungarian version of PROMIS-GH and developed population reference values for its physical and mental health subscales in Hungary. We used both classical test theory and IRT methods to establish the psychometric properties of the measure. PROMIS-GH subscales showed no ceiling and floor effects. All assumptions of IRT (unidimensionality, local independence and monotonicity) were met. Although the Omega Hierarchical value was below the tentative benchmark for GPH, it is important to emphasize that PROMIS-GH is inherently a multidimensional measure, and therefore, individual subscale values within the range of 0.6 and 0.8 seem appropriate both for Omega Hierarchical and ECV [36, 37]. The goodness of fit to the graded response model was acceptable with a few items misfitting. We found no measurement invariance for any sociodemographic characteristics. Strong correlations were found between corresponding PROMIS-GH subscales and SF-36 physical and mental health composite scores. Mean GPH and GMH T-scores in the Hungarian general population were 49.0 and 47.7, respectively.

It is worthwhile to compare our findings about the psychometric performance of PROMIS-GH to those of earlier psychometric studies among members of the general population in the Netherlands and the US [10, 23]. First, unidimensionality was supported with negligible deviations in each study. No local dependence was detected in the Hungarian and Dutch general population samples. The coefficients of the Mokken scale analysis showed that monotonicity was supported in the Hungarian and Dutch samples, and an interesting similarity occurred that in both studies the Global06 item had the smallest distance between the thresholds (Hungarian: − 2.879 to − 0.252; Dutch: − 2.668 to − 0.055). The range of item difficulty values (b) were very similar in all three general population studies with small differences at both ends (US: − 3.0 to 1.5, Hungarian: − 3.7 to 1.7, Dutch: − 3.7 to 1.9) [10, 23]. Ranges of item discrimination parameters (a) were similar for both subscales with slight differences between the US and Dutch studies [10, 23]. While the item discrimination parameters of the Hungarian GPH were in the same range (from 1.6 to 2.3) as the previous two, the Hungarian GMH was somewhat biased due to Global04 (from 1.7 to 8.0), as it usually ranges between 0.5 and 2.5 [31].

The Hungarian overall mean GPH and GMH T-scores (49.0 and 47.7) were slightly lower than those of the US reference population values (GPH: 50.0, GMH: 50.0) and higher than the Dutch values (GPH: 45.2, GMH: 44.7), suggesting that the Hungarian general population is in a better health status than the Dutch (Online Resource 2). By contrast, the standardized Dutch SF-36 physical (49.7) and mental health composite score (52.1) were somewhat higher than the Hungarian scores (48.3 and 48.2), implying that the Dutch general population is in a better health status [51]. However, the Dutch population norm data were collected using the SF-12 and in 1996, which may limit the comparison [52]. A similar pattern was observed for GPH and GMH in the Hungarian general population as in the US and Dutch samples, with a decreasing mean T-score with age, and males reporting better health status than females [25, 53]. However, it should be noted that the US sample (data collected in 2006–2007) and the Dutch sample (data collected in 2016) were obtained considerably earlier compared to this study. In addition, the US calibration sample may not be representative for the European populations. Ultimately, the following characteristics were associated with better physical and mental health in the Hungarian sample: being younger, male, having higher level of education, living in towns, student status, having a higher level of income, having no chronic diseases and reporting better self-perceived health on the first question of the SF-36.

A surprising finding of this study is that the Hungarian general population reported better overall health status than the Dutch general population. Life expectancy in the Netherlands is almost one year higher (81.5) than the weighted EU average (80.6), while life expectancy in Hungary is almost five years (75.7) behind the weighted EU average [54]. In terms of government funding, compulsory and voluntary health insurance and out-of-pocket payments, the Netherlands has one of the highest per capita spending on healthcare in the EU, while Hungary continues to fall behind the EU average in this regard. The greatest contrast might be in the fact that in 2019, 75% of the Dutch general public reported that they were in good health, and this figure did not reach 60% in Hungary in the same year [54]. However, the comparison of PROMIS-GH scores between these two countries is limited by the fact that the Dutch sample was not representative for some important sociodemographic and health-related characteristics of the general population, such as employment and marital status, income and the prevalence of chronic diseases [25].

This study has a few limitations. Our data were collected during the pandemic that might have influenced health status of the general population. However, a recent study has shown that the COVID-19 pandemic had negligible impact on the health status of US patients measured by PROMIS-GH [55]. Furthermore, self-reported health status on the first question of SF-36 in our study was very similar to what had been reported in a pre-COVID online general population survey in Hungary in 2019 [56]. Selection bias might have occurred as online panel data collections may be subject to possible self-selection and underrepresentation of certain groups (e.g. those without internet access) [57]. Another limitation is the cross-sectional nature of this study that prevented us from assessing test–retest reliability and responsiveness of PROMIS-GH.

In conclusion, this study provided an extensive psychometric analysis of the Hungarian PROMIS-GH in a large general population sample and established general population reference values for Hungary. Future research is recommended to replicate this general population study after the COVID-19 pandemic and further test psychometric properties of the Hungarian PROMIS-GH in paper-and-pencil surveys, longitudinal studies and with various patient populations.

### Supplementary Information

Below is the link to the electronic supplementary material.

Supplementary file1 (DOCX 26 KB)

## Data Availability

Data are available from the corresponding author upon a reasonable request.

## References

[1] Nelson EC, Eftimovska E, Lind C, Hager A, Wasson JH, Lindblad S (2015). Patient reported outcome measures in practice. BMJ: Br. Med. J..

[2] Patrick DL, Deyo RA (1989). Generic and disease-specific measures in assessing health status and quality of life. Med. Care.

[3] EuroQol Group: EuroQol—a new facility for the measurement of health-related quality of life. Health Policy **16**(3), 199–208 (1990) 10.1016/0168-8510(90)90421-9 10109801

[4] Ware Jr, J.E., Sherbourne, C.D.: The MOS 36-item short-form health survey (SF-36): I. conceptual framework and item selection. Med. Care **30**(6), 473–483 (1992) 1593914

[5] Cella D, Yount S, Rothrock N, Gershon R, Cook K, Reeve B (2007). The patient-reported outcomes measurement information system (PROMIS): progress of an NIH Roadmap cooperative group during its first two years. Med. Care.

[6] Cella D, Riley W, Stone A, Rothrock N, Reeve B, Yount S (2010). The patient-reported outcomes measurement information system (PROMIS) developed and tested its first wave of adult self-reported health outcome item banks: 2005–2008. J. Clin. Epidemiol..

[7] Cella D, Choi SW, Condon DM, Schalet B, Hays RD, Rothrock NE (2019). PROMIS® adult health profiles: efficient short-form measures of seven health domains. Value Health.

[8] Hays RD, Morales LS, Reise SP (2000). Item response theory and health outcomes measurement in the 21st century. Med. Care.

[9] Reeve, B.B., Hays, R.D., Bjorner, J.B., Cook, K.F., Crane, P.K., Teresi, J.A., et al.: Psychometric evaluation and calibration of health-related quality of life item banks: plans for the patient-reported outcomes measurement information system (PROMIS). Med. Care **45**(5 Suppl 1), S22–S31 (2007) 10.1097/01.mlr.0000250483.85507.04 17443115

[10] Hays RD, Bjorner JB, Revicki DA, Spritzer KL, Cella D (2009). Development of physical and mental health summary scores from the patient-reported outcomes measurement information system (PROMIS) global items. Qual. Life Res..

[11] Katzan IL, Lapin B (2018). PROMIS GH (patient-reported outcomes measurement information system global health) scale in stroke: a validation study. Stroke.

[12] Suriani RJ, Kassam HF, Passarelli NR, Esparza R, Kovacevic D (2020). Validation of PROMIS Global-10 compared with legacy instruments in patients with shoulder instability. Shoulder Elbow.

[13] Lapin B, Davin S, Stilphen M, Benzel E, Katzan IL (2020). Validation of PROMIS CATs and PROMIS global health in an interdisciplinary pain program for patients with chronic low back pain. Spine.

[14] Kahan JB, Kassam HF, Nicholson AD, Saad MA, Kovacevic D (2019). Performance of PROMIS global-10 to legacy instruments in patients with lateral epicondylitis. Arthroscopy.

[15] Nicholson AD, Kassam HF, Pan SD, Berman JE, Blaine TA, Kovacevic D (2019). Performance of PROMIS global-10 compared with legacy instruments for rotator cuff disease. Am. J. Sports Med..

[16] Parker DJ, Werth PM, Christensen DD, Jevsevar DS (2022). Differential item functioning to validate setting of delivery compatibility in PROMIS-global health. Qual. Life Res..

[17] D'Souza A, Magnus BE, Myers J, Dispenzieri A, Flynn KE (2020). The use of PROMIS patient-reported outcomes (PROs) to inform light chain (AL) amyloid disease severity at diagnosis. Amyloid.

[18] IsHak WW, Pan D, Steiner AJ, Feldman E, Mann A, Mirocha J (2017). Patient-reported outcomes of quality of life, functioning, and GI/psychiatric symptom severity in patients with inflammatory bowel disease (IBD). Inflamm. Bowel Dis..

[19] Slavin V, Gamble J, Creedy DK, Fenwick J, Pallant J (2019). Measuring physical and mental health during pregnancy and postpartum in an Australian childbearing population - validation of the PROMIS global short form. BMC Pregnancy Childbirth.

[20] Allen J, Alpass FM, Stephens CV (2018). The sensitivity of the MOS SF-12 and PROMIS® global summary scores to adverse health events in an older cohort. Qual. Life Res..

[21] Shim J, Hamilton DF (2019). Comparative responsiveness of the PROMIS-10 global health and EQ-5D questionnaires in patients undergoing total knee arthroplasty. Bone Joint J..

[22] Philipp R, Lebherz L, Thomalla G, Härter M, Appelbohm H, Frese M (2021). Psychometric properties of a patient-reported outcome set in acute stroke patients. Brain Behav..

[23] Pellicciari L, Chiarotto A, Giusti E, Crins MHP, Roorda LD, Terwee CB (2021). Psychometric properties of the patient-reported outcomes measurement information system scale v.12: global health (PROMIS-GH) in a Dutch general population. Health Qual. Life Outcomes.

[24] Liu H, Cella D, Gershon R, Shen J, Morales LS, Riley W (2010). Representativeness of the patient-reported outcomes measurement information system internet panel. J. Clin. Epidemiol..

[25] Elsman EBM, Roorda LD, Crins MHP, Boers M, Terwee CB (2021). Dutch reference values for the patient-reported outcomes measurement information system scale v.12 - global health (PROMIS-GH). J. Patient-Report. Outcomes.

[26] Rencz, F., Janssen, M.F.: Analyzing the pain/discomfort and anxiety/depression composite domains and the meaning of discomfort in the EQ-5D: a mixed-methods study. Value Health **25**(12), 2003–2016 (2022) 10.1016/j.jval.2022.06.012 35973925

[27] Rencz, F., Brodszky, V., Janssen, M.F.: A direct comparison of the measurement properties of EQ-5D-5L, PROMIS-29+2 and PROMIS Global Health instruments and EQ-5D-5L and PROPr utilities in a general population sample. Value Health (2023). 10.1016/j.jval.2023.02.002 10.1016/j.jval.2023.02.002 36804583

[28] Jenei, B., Bató, A., Mitev, A.Z., Brodszky, V., Rencz, F.: Hungarian PROMIS-29+2: psychometric properties and population reference values. Qual. Life Res. (2023). 10.1007/s11136-023-03364-7 10.1007/s11136-023-03364-7 PMC9931172 36792819

[29] HealthMeasures (2017). PROMIS global health scoring manual. http://www.healthmeasures.net/images/PROMIS/manuals/PROMIS_Global_Scoring_Manual.pdf. Accessed 7 Sept 2021

[30] Brazier JE, Harper R, Jones N, O'cathain A, Thomas K, Usherwood T (1992). Validating the SF-36 health survey questionnaire: new outcome measure for primary care. BMJ.

[31] Reeve BB, Fayers P (2005). Applying item response theory modeling for evaluating questionnaire item and scale properties. Assess. Qual. Life Clin Trials: Methods Pract..

[32] Terwee CB, Bot SDM, de Boer MR, van der Windt DAWM, Knol DL, Dekker J (2007). Quality criteria were proposed for measurement properties of health status questionnaires. J. Clin. Epidemiol..

[33] Rosseel, Y.: lavaan: an R package for structural equation modeling. J. Stat. Softw. **48**(2), 1–36 (2012)

[34] Hu LT, Bentler PM (1999). Cutoff criteria for fit indexes in covariance structure analysis: Conventional criteria versus new alternatives. Struct. Equ. Model.: A Multidiscip. J..

[35] Rosseel Y (2012). Lavaan: an R package for structural equation modeling and more. Version 0.5–12 (BETA). J. Stat. Softw..

[36] Reise SP, Scheines R, Widaman KF, Haviland MG (2013). Multidimensionality and structural coefficient bias in structural equation modeling: a bifactor perspective. Educ. Psychol. Measur..

[37] Rodriguez A, Reise SP, Haviland MG (2016). Applying bifactor statistical indices in the evaluation of psychological measures. J. Pers. Assess..

[38] Revelle, W.R.: psych: Procedures for psychological, psychometric, and personality research. Northwestern University, Evanston, Illinois. R package version 2.3.3 (2023). https://CRAN.R-project.org/package=psych. Accessed 17 Jun 2023

[39] Mokken, R.J.: A theory and procedure of scale analysis: with applications in political research: De Gruyter Mouton. ISBN: 9783110813203 (2011)

[40] van der Ark LA (2007). Mokken scale analysis in R. J. Stat. Softw..

[41] Chalmers RP (2012). mirt: a multidimensional item response theory package for the R environment. J. Stat. Softw..

[42] Samejima, F.: Estimation of latent ability using a response pattern of graded scores. Psychometrika Monogr. Suppl. **34**(4, Pt. 2), 100 (1969)

[43] Kang T, Chen TT (2011). Performance of the generalized S-X2 item fit index for the graded response model. Asia Pac. Educ. Rev..

[44] Choi SW, Gibbons LE, Crane PK (2011). Lordif: an R package for detecting differential item functioning using iterative hybrid ordinal logistic regression/item response theory and Monte Carlo simulations. J. Stat. Softw..

[45] Crane PK, Gibbons LE, Jolley L, van Belle G (2006). Differential item functioning analysis with ordinal logistic regression techniques: DIFdetect and difwithpar. Med. Care.

[46] Kopf J, Zeileis A, Strobl C (2015). Anchor selection strategies for DIF analysis: review, assessment, and new approaches. Educ. Psychol. Measur..

[47] Swinscow, T.D.V., Campbell, M.J. (2002). Statistics at square one: Bmj London. 0727915525

[48] Hungarian central statistical office (2016). Microcensus 2016. Available from: https://www.ksh.hu/docs/eng/xftp/idoszaki/microcensus2016/microcensus_2016_3.pdf. Accessed 7 Sept 2021.

[49] Oosterveer, D.M., Arwert, H., Terwee, C.B., Schoones, J.W., Vlieland, T.P.M.V.: Measurement properties and interpretability of the PROMIS item banks in stroke patients: a systematic review. Qual. Life Res. **31**(12), 3305–3315 (2022) 10.1007/s11136-022-03149-4 35567674

[50] Szende A, Németh R (2003). Health-related quality of life of the Hungarian population. Orv. Hetil..

[51] Gandek B, Ware JE, Aaronson NK, Apolone G, Bjorner JB, Brazier JE (1998). Cross-validation of item selection and scoring for the SF-12 health survey in nine countries: results from the IQOLA project. J. Clin. Epidemiol..

[52] Gandek B, Ware JE (1998). Methods for validating and norming translations of health status questionnaires: the IQOLA project approach. J. Clin. Epidemiol..

[53] HealthMeasures (2021). PROMIS score cut points. https://www.healthmeasures.net/score-and-interpret/interpret-scores/promis/promis-score-cut-points, Accessed 7 Sept 2021

[54] OECD: Health at a Glance 2021: OECD Indicators. OECD Publishing, Paris (2021). 10.1787/ae3016b9-en

[55] Lapin BR, Tang WHW, Honomichl R, Hogue O, Katzan IL (2021). Evidence of stability in patient-reported global health during the COVID-19 Pandemic. Value Health.

[56] Rencz F, Tamási B, Brodszky V, Ruzsa G, Gulácsi L, Péntek M (2020). Did You get what you wanted? Patient satisfaction and congruence between preferred and perceived roles in medical decision making in a hungarian national survey. Value Health Reg. Issues.

[57] Bethlehem J (2010). Selection bias in web surveys. Int. Stat. Rev..

