# Supplementary material for: Psychometric properties and general population reference values for PROMIS Global Health in Hungary
Source: Eur J Health Econ. 2023 Jun 28;25(4):549–62. doi: 10.1007/s10198-023-01610-w (PMC11136746; doi:10.1007/s10198-023-01610-w)
Supplement: Supplementary file 1 — Supplementary file1 (DOCX 26 KB) [file 10198_2023_1610_MOESM1_ESM.docx]

**Supplementary Information**

**Psychometric properties and general population reference values for PROMIS Global Health in Hungary**

Alex Bató, Valentin Brodszky, Ariel Zoltán Mitev, Balázs Jenei, Fanni Rencz

**Correspondence:**

Fanni Rencz

Department of Health Policy

Corvinus University of Budapest

Address: 8 Fővám tér, H-1093 Budapest, Hungary

E-mail: fanni.rencz@uni-corvinus.hu

**Online Resource 1** Residual correlations from the confirmatory factor analysis

**Online Resource 2** Comparison of PROMIS Global Physical Health and Global Mental Health mean T-scores across the Hungarian, Dutch and US general population

**Online Resource 1 Residual correlations from the confirmatory factor analysis**

| **Global Physical Health items** | | | | |
| --- | --- | --- | --- | --- |
| Items | Global03 | Global06 | Global07 | Global08 |
| Global03 | 0.000 | - | - | - |
| Global06 | 0.058 | 0.000 | - | - |
| Global07 | -0.033 | -0.021 | 0.000 | - |
| Global08 | -0.029 | -0.071 | 0.066 | 0.000 |
| **Global Mental Health items** | | | | |
| Items | Global02 | Global04 | Global05 | Global10 |
| Global02 | 0.000 | - | - | - |
| Global04 | -0.004 | 0.000 | - | - |
| Global05 | 0.026 | -0.006 | 0.000 | - |
| Global10 | -0.067 | 0.029 | -0.013 | 0.000 |

Global02 = quality of life, Global03 = physical health, Global04 = mental health, Global05 = satisfaction with discretionary social activities, Global06 = physical function, Global07 = pain (reverse coded 5-level item), Global08 = fatigue, Global10 = emotional problems

**Online Resource 2 Comparison of PROMIS Global Physical Health and Global Mental Health mean T-scores across the Hungarian, Dutch and US general population**

|  | **Weighted Hungarian population (N, %)** | **Dutch population (N, %)^a^** | **Global Physical Health** | | | | **Global Mental Health** | | | |
| --- | --- | --- | --- | --- | --- | --- | --- | --- | --- | --- |
|  |  |  | **US population (N, %)^b^** | **Hungarian mean T-score (SD)** | **Dutch mean T-score (SD)** | **US mean T-score (SD)** | **US population (N, %)** | **Hungarian mean T-score (SD)** | **Dutch mean T-score (SD)** | **US mean T-score (SD)** |
| Total | 1700 (100) | 4370 (100) | 5228 (100) | 49.0 (9.1) | 45.2 (9.2) | 50.0 (10.0) | 5215 (100) | 47.7 (9.5) | 44.7 (8.0) | 50.0 (10.0) |
| Gender | | | | | | | | | | |
| Female | 902 (53) | 2301 (53) | 3015 (58) | 47.8 (9.2) | 44.5 (9.1) | 49.1 (10.1) | 3008 (58) | 46.4 (9.4) | 44.1 (8.0) | 49.4 (10.0) |
| Male | 798 (47) | 2069 (47) | 2212 (42) | 50.5 (8.8) | 46.1 (9.2) | 51.2 (9.8) | 2206 (42) | 49.3 (9.4) | 45.5 (8.0) | 50.8 (10.0) |
| Age groups (years) | | | | | | | | | | |
| 18-34 | 428 (25.2) | 891 (20) | 1182 (23) | 51.5 (8.2) | 47.8 (8.0) | 51.6 (8.4) | 1183 (23) | 49.5 (10.2) | 45.6 (8.0) | 48.5 (9.7) |
| 35-44 | 331 (19.5) | 753 (17) | 865 (17) | 50.0 (8.7) | 45.2 (8.2) | 50.1 (9.8) | 863 (17) | 48.2 (10.0) | 43.8 (8.3) | 48.4 (10.4) |
| 45-54 | 272 (16.0) | 646 (15) | 910 (17) | 48.7 (9.2) | 44.6 (9.3) | 48.2 (10.9) | 902 (17) | 47.2 (9.4) | 43.6 (8.1) | 48.2 (10.3) |
| 55-64 | 286 (16.8) | 918 (21) | 875 (17) | 47.7 (9.6) | 43.4 (9.7) | 48.8 (11.3) | 873 (17) | 46.3 (8.9) | 43.6 (8.0) | 50.3 (10.5) |
| 65-74 | 329 (19.4) | 893 (20) | 713 (14) | 47.1 (9.3) | 45.1 (9.5) | 51.0 (9.9) | 715 (14) | 46.8 (8.4) | 45.9 (7.4) | 53.1 (8.8) |
| 75+ | 54 (3.2) | 269 (6) | 683 (13) | 45.2 (7.7) | 44.9 (9.8) | 49.9 (9.2) | 679 (13) | 46.8 (8.4) | 47.3 (7.7) | 53.4 (8.4) |

Hungarian mean T-scores were age- and gender-weighted. SD = standard deviation

a: Elsman, E.B.M., Roorda, L.D., Crins, M.H.P., Boers, M., Terwee, C.B.: Dutch reference values for the patient-reported outcomes measurement information system scale v.12 - global health (PROMIS-GH). J. Patient-Report. Outcomes **5**(1), 38 (2021)

b: HealthMeasures (2021). PROMIS Score Cut-Points. http://www.healthmeasures.net/score-and-interpret/interpret-scores/promis/promis-score-cut-points, Accessed 7 September 2021
